# Use of TaqMan Array Card for the Detection of Respiratory Viral Pathogens in Children under 5 Years Old Hospitalised with Acute Medical Illness in Ballabgarh, Haryana, India

**DOI:** 10.4103/ijmm.IJMM_18_146

**Published:** 2019

**Authors:** Bharti Gaur, Siddhartha Saha, A. Danielle Iuliano, Sanjay K. Rai, Anand Krishnan, Seema Jain, Brett Whitaker, Jonas Winchell, Renu B. Lal, Shobha Broor

**Affiliations:** ** Department of Microbiology, All India Institute of Medical Sciences, New Delhi, India; 1 Influenza Division, Centers for Disease Control and Prevention, U.S Embassy, New Delhi, India; 2 Influenza Division, Centers for Disease Control and Prevention, Atlanta, Georgia, USA; 3 Centre for Community Medicine, All India Institute of Medical Sciences, New Delhi, India; 4 Division of Viral Diseases, Centers for Disease Control and Prevention, Atlanta, Georgia, USA; 5 Division of Bacterial Diseases, Centers for Disease Control and Prevention, Atlanta, Georgia, USA

**Keywords:** Micro-fluidic card, sensitivity, specificity, viral pathogen diagnosis

## Abstract

Historical specimens collected from hospitalized children were tested for the following 13 viruses: influenza A and B; respiratory syncytial virus (RSV); parainfluenza viruses 1–3; human metapneumovirus; rhinovirus; coronaviruses 229E, OC43, NL63 and HKU1 and Adenovirus using monoplex real-time reverse transcriptase polymerase chain reaction (rRT-PCR). They were retested using TaqMan Array Card (TAC), a micro-fluidic system, capable of simultaneous multi-pathogen testing, to evaluate its sensitivity and specificity against monoplex rRT-PCR. TAC showed high sensitivity (71%–100%) and specificity (98%–100%) for these viruses in comparison to monoplex rRT-PCR. Multi-specimen detection with high sensitivity and specificity makes TAC a potentially useful tool for both surveillance and outbreak investigations.

## Introduction

The TaqMan array card (TAC) (Thermo Fisher, Carlsbad CA), a real-time reverse transcriptase polymerase chain reaction (rRT-PCR) platform in a 384-well micro-fluidic card format, is one of the available platforms, which allows rapid and simultaneous detection of multiple pathogens. TAC utilises rRT-PCR assays that are pre-spotted and dried in each individual well according to the end user’s design layout.^[1,2]^

TAC has been studied for its application for infectious diseases, especially outbreak investigations by the U.S. Centers for Disease Control and Prevention (CDC).^[3,4]^ However, studies in different settings, including low- and middle-income countries, are limited.^[5]^ We compared the sensitivity and specificity of TAC with monoplex rRT-PCR performed in a 96-well PCR plate format as reference for the detection of 13 common respiratory viruses.

## Materials And Methods

### Study design and specimens selection for TaqMan Array card testing

Nasal and throat specimens (only nasal swabs from infants) were collected from the hospitalised children with any acute medical illness within 24 hours of admission using polyester swabs (Thermo Fisher Inc., China),^[6]^ to test for respiratory pathogens using rRT-PCR; study details were published previously.^[7]^ Of the 298 previously tested samples, 135 specimens were selected, which were either positive for at least one pathogen by rRT-PCR or negative for all viruses tested, had been subjected to no more than two freeze-thaw cycles, had cycle threshold (Ct) values (18–38) and had sufficient sample volume (>200 μl).

### Molecular testing of viruses using real-time reverse transcriptase polymerase chain reaction and TaqMan Array card

The steps involved in both the methods are compared in Table 1. The TAC used in this study was designed to test six specimens and included negative temperature coefficient, positive temperature coefficient, internal plate control and a panel of 31 monoplex rRT-PCR assays for 16 viral and 14 bacterial targets. A total volume of 100 μl of master-mix and total nucleic acid (TNA) was loaded. Final volume of master-mix contained similar concentration of primer and probe as in rRT-PCR. Primers and probes used were designed and synthesised at CDC.^[2,8]^ Clinical and analytical validations for each assay (sensitivity, specificity and limit of detection) have also been determined at CDC.^[2]^

The reaction mixes for each specimen were prepared in a clean assay setup room, using AgPath-ID One-step RT-PCR kits as per the CDC protocol.^[2]^ All TAC runs were performed on the Thermo Fisher Scientific Inc., ViiA™ 7 real-time PCR instrument using the AgPath-ID One-Step RT-PCR kit (Thermo Fisher Scientific Inc., Foster City, CA). A Ct value of 43 was considered as cut-off value for interpretation as positive or negative for both the methods.

### Statistical analysis

Sensitivity and specificity of the TAC for different pathogens were analysed using Stata 12 (StataCorp LP version 12, College Station, Texas, US) with monoplex rRT-PCR results as reference. The concordance between TAC and rRT-PCR for each pathogen in the same specimen was assessed using Cohen’s kappa coefficient.^[9]^

## Results

As summarised in Table 1, using monoplex rRT-PCR, the total time was 9–10 h, whereas TAC required ~3–4 h to complete the testing. Of 135 specimens tested using monoplex rRT-PCR assays, 85 (63%) specimens were found positive including nine specimens with co-detections, whereas 81 (60%) specimens were found positive including eight co-detections using TAC. Using monoplex rRT-PCR, respiratory syncytial virus (RSV) (33, 24%) was most commonly detected, followed by rhinovirus (Rhiv) (16, 12%); influenza B (8, 6%) and influenza A, adenovirus (AdV), parainfluenza viruses (PIV), human metapneumovirus (hMPV) and coronaviruses (CoV) (7 each, 5%). Using TAC, RSV remained the most commonly detected virus (29, 22%), followed by Rhiv (16, 12%), influenza A and B viruses (8 each, 6%), AdV (7, 5%), PIV (7, 5%), hMPV (6, 4%) and CoV (6, 4%).

**Figure 1:**
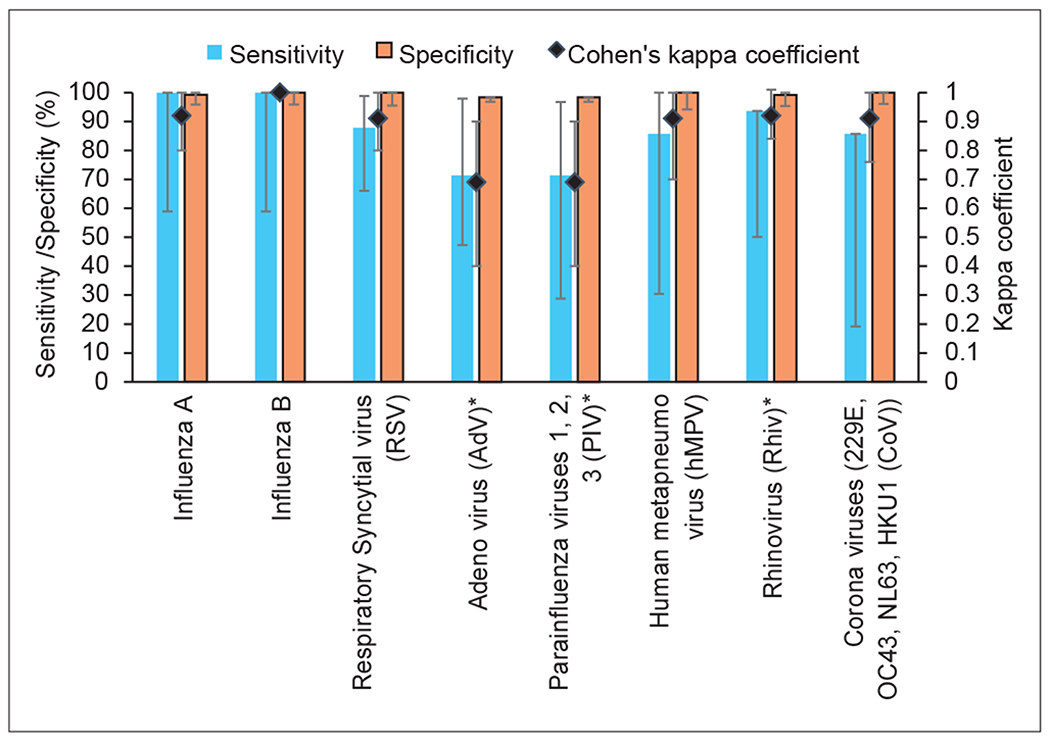
Sensitivity, specificity and Cohen’s Kappa coefficient for the detection of selected respiratory viruses using TaqMan Array card compared to real-time reverse transcriptase polymerase chain reaction. *Note: For some viruses, monoplex real-time reverse transcriptase polymerase chain reaction and TaqMan Array card identified different specimens as positive, so the sensitivity and specificity are not 100%

In comparison to rRT-PCR, sensitivity and specificity of TAC ranged between 72%–100% and 98%–100%, respectively [Figure 1]. The highest sensitivity of TAC was for influenza A and B viruses (100%) and lowest for PIV (71%) and AdV (71%). Positive predictive value was highest for influenza B, RSV, hMPV and CoV (each 100%) and lowest for AdV (71%) and PIV (71%) using the TAC assay. RSV (96%) had the lowest negative predictive value, but all negative predictive values were relatively high (98%–100%). Cohen’s kappa coefficient ranged from 0.69 to 1.0 for different pathogens and was lowest for AdV (0.69, 95% confidence interval [CI]: 0.4–0.9) and PIV (0.69, 95% CI: 0.4–0.9) and highest for influenza B virus (1.0, one-sided CI: 1.0).

## Discussion

In this study, we observed that TAC offers several advantages over monoplex rRT-PCR while maintaining high sensitivity (71%–100%) and specificity (98%–100%) for the respiratory pathogen assays assessed similar to earlier studies.^[1,2]^ Advantages of the TAC included a simple setup, lower specimen volume requirement, efficient consumption of TNA extracts and rRT-PCR reaction reagents and rapid, simultaneous detection of multiple pathogens in a single experiment. The TAC format due to its closed system design requiring less handling of assay and primers/probes minimises the risk for user error and cross-contamination besides requiring fewer tubes.^[4,10,11]^ Furthermore, the TAC can be customised with different assays and configurations, unlike other commercial multi-pathogen detection platforms, which is useful in research settings, conducting disease surveillance or investigating outbreaks of unknown aetiology.^[4,8]^ Conventional monoplex rRT-PCR assays for the detection of respiratory viruses can be tedious, expensive and require large volumes of biological specimens.^[2,12]^ In comparison with monoplex rRT-PCR assays, TAC required 1/3 times less volume of TNA for detecting the same number of pathogens.^[8,12]^

While the kappa statistic indicated substantial to perfect agreement for all viral pathogens, assays for AdV and PIV showed lower agreement between the two methods (kappa 0.69) compared to assays for the other respiratory viruses. This could be due to several factors such as, but not limited to, assay optimisation on different annealing temperatures and evaporation of reaction volume from incomplete card sealing, which can cause fluorescent anomalies or probe instability. Furthermore, the freeze-thaw degradation of specimens for retesting via TAC may have led to false-negative results.

One of the limitations of our study was wide CIs around the point estimates for TAC sensitivity for CoV, hMPV and PIV, because of few positive samples. Some viruses were not very prevalent, including PIV and CoV, making it difficult to interpret their sensitivity and specificity. We could test for 135 specimens out of 298 because of limited number of cards available for this study.

In spite of higher throughput of TAC card than rRT-PCR when used for multiple pathogens, its use in low-resource settings may not yet be feasible because of the high cost of ViiA™ 7 machine. Further, in case of failure of an assay for even one target pathogen, the entire set of targets in the card have to be repeated, unlike in monoplex, where individual wet assay can be performed for failed targets, which also makes TAC expensive.

In spite of these limitations, our study demonstrated that TAC results were comparable to monoplex rRT-PCR testing, but took less time for simultaneous detection of common respiratory viruses which might be useful during outbreak investigations.

## Figures and Tables

**Table 1:** Comparison of methods for TaqMan Array Card assays with monoplex real-time reverse transcriptase polymerase chain reaction assays for respiratory viral pathogens

Molecular tools	rRTPCR	TAC
Extraction platform	Roche MagNA Pure LC 2.0 (Roche, Indianapolis, US)	Magna Pure Compact (Roche Diagnostics, Indianapolis, USA)
Extraction protocol	100 μL TNA was extracted from 100 μL of clinical specimen as per manufacturer’s instructions	100 μL TNA was extracted from 100 μL of stored clinical specimen as per manufacturer’s instructions
Primer and probe used	Primers and probes used, were designed and synthesised at CDC	Primers and probes used, were designed and synthesised at CDC6. Clinical and analytical validations for each assay (sensitivity, specificity and LOD) has also been determined at CDC^[2]^
PCR cycling conditions	The thermo-cycling conditions for influenza A and B assays were: 50°C for 30 min, 95°C for 10 min, and 45 cycles of 95°C for 15 s, and 55°C for 30 s with data collection. For other viral assays, the thermo-cycling conditions were: 45°C for 10 min, 94°C for 10 min, and 45 cycles of 94°C for 30 s followed by 60°C for 1 min with data collection	Cycling conditions on the ViiA™ 7 for TACs were 45°C for 10 min, 95°C for 10 min, followed by 45 two-step cycles of 95°C for 20 s and 60°C for 1 min with data collection
Interpretation of test	A Ct cut-off value of 43 was established and Ct values were interpreted as follows: positive results included Ct values <43, negative results included Ct values ≥43, and samples with no amplification detected were considered negative	A Ct cut-off value of 43 was established and Ct values were interpreted as follows: positive results included Ct values <43, negative results included Ct values ≥43, and samples with no amplification detected were considered negative
Time taken to perform the complete run	9-10 h	3-4 h

rRT PCR: Real-time reverse transcriptase polymerase chain reaction, TNA: Total nucleic acid, TAC: TaqMan Array card, LOD: Limit of detection, CDC: Centers for disease control and prevention, Ct: Cycle threshold
